# Harnessing Ethnographic Methods to Explore the Epidemiological Significance of Livestock Trading Practices: The Case of Chennai's Broiler Chicken Trade

**DOI:** 10.1155/tbed/7965056

**Published:** 2025-10-09

**Authors:** Ivo Syndicus, Vigneshvaran Paramasivam, Ganesh Janarthanan, Eve Houghton, Pallavi Mishra, Kavita Yadav, Vimal Rajkumar Nallathambi, Rajib Dasgupta, Guillaume Fournié, Kumaravel Papaiyan, Tony Barnett

**Affiliations:** ^1^Veterinary Epidemiology, Economics and Public Health Group, Department of Pathobiology and Population Sciences, The Royal Veterinary College, University of London, Hatfield, UK; ^2^Department of Microbiology, Madras Veterinary College, Tamil Nadu Veterinary and Animal Sciences University, Chennai, India; ^3^Centre of Social Medicine and Community Health, School of Social Sciences, Jawaharlal Nehru University, New Delhi, India; ^4^George Institute for Global Health, New Delhi, India; ^5^Department of Veterinary and Animal Husbandry Extension Education, Madras Veterinary College, Tamil Nadu Veterinary and Animal Sciences University, Chennai, India; ^6^INRAE, VetAgro Sup, UMR EPIA, Université de Lyon, Marcy l'Etoile, France; ^7^INRAE, VetAgro Sup, UMR EPIA, Université Clermont Auvergne, Saint Genès Champanelle, France; ^8^Veterinary College and Research Institute, Tamil Nadu Veterinary and Animal Sciences University, Udumalpet, India; ^9^The Firoz Lalji Centre for Africa, London School of Economics and Political Science, London, UK

## Abstract

Compared to the practices in production and retailing of broiler chickens, the wholesale broiler trade is less well understood. With the increasing concern about zoonotic diseases with pandemic potential transmissible by poultry, especially high pathogenicity avian influenza, it is important to understand how and through what kinds of business models and practices broilers are moved between production areas and cities. Such understanding can contribute to establish tailored surveillance and mitigation approaches. Ethnographic methods are uniquely positioned for gaining in-depth insights into human practices and the determinants that underpin their persistence and motivate or constrain their change. Ethnography's potential to contribute to epidemiological risk modelling and dynamic approaches to risk prevention and mitigation, for example through exploring biosecurity practices and the socio-economic factors influencing them, has been underutilised to date. In this study we took an ethnographic approach to research with wholesale live broiler traders in the South Indian city of Chennai, visiting premises and conducting interviews with interlocutors of 13 broiler wholesale enterprises and some related upstream and downstream actors over a period of 18 months in 2021 and 2022. We describe the business relations between wholesale broiler traders and upstream actors in production whom traders source birds from and their downstream wholesale clients such as retailers, caterers and restaurants. We then discuss traders' business models and associated broiler procurement and distribution practices from a perspective of disease transmission risks. Further, we show how situating current configurations of wholesale broiler trade within its broader historical trajectory and anticipated future from a Chennai-centric perspective constitutes important context towards dynamic approaches to assess evolving disease transmission risks in changing poultry production and distribution networks (PDNs). More broadly, the study thus proposes methodological innovations for epidemiology and One Health research by further embedding ethnography and social sciences into their toolkits.

## 1. Introduction

Chickens play an important role in global concerns about zoonotic disease risks emanating from livestock systems. They commonly carry food-borne pathogens such as campylobacter and salmonella, may spread associated antimicrobial resistances [1–4] and are drawing significant attention in relation to transmission of avian influenza viruses [5, 6]. Zoonotic disease risks emerge along different configurations of chicken production and distribution networks (PDNs) [7]—that is the network of actors and sites involved in production, transport, slaughter, processing, retail—and further in food preparation and consumption. Besides the upstream processes of production on farms and the downstream handling during slaughter, processing and retail, the practices and configurations of live poultry transport and trading networks between production and retail constitute an important area for understanding potential disease transmission risks that have so far received less detailed attention [8].

Two main types of study tend to be conducted to assess the epidemiological significance of live poultry trading practices along value chains or trade networks. On the one hand, network analyses relying on data from quantitative surveys are used to describe trade flows and resulting network configurations, and assess their epidemiological significance, especially in relation to understanding potential transmission pathways of avian influenza viruses [9–18]. Their results can help to parameterise models of pathogen transmission [19, 20]. These studies leave fundamental knowledge gaps about how such trade networks come into existence in the first place and what influences their configuration and actors' practices within them—which are important for understanding and anticipating how these networks may change over time. Such considerations are likely to make significant contributions to successful interventions.

In contrast, studies employing qualitative mapping that include trading practices, and their governance, can provide important data to inform risk assessments across value chains [21, 7, 22, 23, 6]. These studies characterise value chains (or PDNs) in terms of their structural configurations to identify factors that could create disease risks. They provide a better understanding about the environment in which such risks are generated and the roles of actors within them, in turn enabling different kinds of insights to inform interventions for limiting disease risks. For example, the distinction of governance typologies, such as buyer-driven or producer-driven value chains [24], and an understanding of the mutual effects and reinforcements between economic drivers and trading practices and configurations [25] are important considerations towards the assessment of how disease risks are generated. In their broad assessment of PDNs through key informants, however, qualitative studies mapping value chains often produce only general characterisations that provide limited insights into the specific roles and practices of actors, the relations and interactions between them, and the broader dynamics such roles, practices and business relations are subject to, especially from the perspective of these actors themselves. The insights into the structural configuration of poultry PDNs and related disease risks that such studies generate, however, can provide a basis for informing more detailed studies, including quantitative network analyses and in-depth qualitative studies on specific PDNs or nodes within them.

A nascent interdisciplinary innovation of more in-depth qualitative research for understanding how actors engage in poultry PDNs that we seek to further advance here is to draw from the toolkit of methods commonly used in social anthropology to address questions relevant to veterinary epidemiology and One Health [26, 27]. This kind of applied social science is using anthropological expertise in research design, data collection and analysis to gain a more in-depth understanding of practices by actors and has previously been applied to zoonotic disease risks emerging from live poultry trade [5, 28]. Such approaches move analysis beyond the ‘what' and ‘how' toward the ‘why' of specific practices and business models and thus of the broader context in which these occur and how they are shaped into distinct configurations of social, cultural and economic relationships. Such approaches enable more detailed insights into the perceptions and practices of actors, and thus how actors react to experienced and anticipated change. Overall, a research process inspired by less structured and more open-ended ethnographic approaches allows for more focused and in-depth empirical insights compared to qualitative value chain mapping approaches and thus also provides a better understanding towards addressing and mitigating zoonotic disease risks. In this study, we further such an agenda through a focus on live broiler wholesalers in Chennai, capital of the South Indian state of Tamil Nadu.

According to the latest Indian livestock census, Tamil Nadu is the state with the highest poultry population in India, and among the top five poultry meat producing states [29]. Chennai is the largest city in Tamil Nadu by far, and most populated metropolitan area in South India, constituting a significant wholesale broiler trading hub. While overall chicken consumption remains low in India by global comparison, it has increased six-fold between 1993/4 and 2011/2—a trend that is expected to continue [30, 31]. Work on chicken consumption in Chennai illustrates how the conjunction of increased availability, accessibility and desirability of chicken meat facilitated its shift from a less valued meat, that is, compared to mutton, to the most consumed one [32].

For understanding the context influencing the epidemiological significance of live broiler trading into Chennai, this study sets out to explore: (1) who are the actors trading in live broiler chickens in Chennai and in their procurement from production areas, (2) how they operate and (3) the historical and current trajectories of their roles as market actors. This comprises wholesalers' business models and the relations they have with other relevant actors in the PDN, such as upstream relations with producers, downstream wholesale clients including retailers, restaurants, canteens and caterers and any business relations or coordination among wholesalers themselves. Broiler wholesalers are usually not limited to wholesale roles but are, to varying degrees, also engaged in retail businesses. More recently some have ventured into production. We are thus also interested in the role of wholesalers in broiler PDN dynamics more broadly, both historically and today—for example, how broiler wholesalers came to constitute specialised enterprises that nevertheless are not necessarily confined to wholesaler roles. These dynamics include how wholesalers experience and respond to business challenges and adapt to changing circumstances, from disruptions such as the COVID-19 pandemic to the broader outlook for the sector and their roles within it.

We first provide (1) a brief history to the wholesale broiler trade in Chennai and (2) a description of the PDN which explores the relations between wholesalers and upstream actors in poultry production, on one hand, and with their downstream clients in retail or catering on the other hand. We then discuss (3) epidemiological considerations resulting from this trade configuration and related practices and conclude with (4) a discussion of the outlook for broiler distribution networks in Chennai and the dynamics of current and anticipated changes.

## 2. Methods

### 2.1. Research Process and Data Collection

This study has been conducted as part of the Global Challenges Research Fund (GCRF) One Health Poultry Hub, a UK-funded development research programme towards addressing zoonotic disease risks in the context of intensifying chicken production in South and Southeast Asia, with Tamil Nadu as one of five focus sites. Initial key informant interviews enabled a general mapping of major poultry PDNs in Tamil Nadu. This initial mapping exercise identified some key areas of potential zoonotic transmission risks and pointed us to the potential epidemiological importance of the continued growth and increasing intensification of the broiler sector in Tamil Nadu, and, within it, of the relatively little understood wholesale trade segment.

The research process for this study then followed an ethnographic approach [33] to engagement with interlocutors and to learning about their roles and practices. This mainly comprised of a gradual process of building rapport with interlocutors through repeated contact and visits to their offices, wholesale trading premises and retail outlets. These processes of building rapport and spending time in wholesale and retail premises including the informal conversations that these entail—what ethnographers refer to as ‘participant observation' [33]—were complemented with more formal semi-structured interviews. In contrast to other qualitative approaches, for example value chain mapping approaches that rely on key informant interviews, an ethnographic approach allows for a more context-sensitive and in-depth understanding of practices through its grounding in empirical insights of what people do within their specific settings. Spending time with interlocutors in their own work environments on a regular or recurrent basis facilitates both an understanding of cumulative depth and detail as much as it can open pathways to change initially gained understandings through deeper insights into actual practices or complex issues that may be omitted in singular interviews aimed at a general understanding.

Primary data collection was conducted intermittently over 18 months between March 2021 and September 2022. Our sampling strategy aimed to capture variations in business models and thereby cover the range of wholesale trading configurations in Chennai as widely as possible. We started the research process through a focused ethnographic engagement with two Chennai-based poultry retailers over a period of 4 months. These retailers were selected based on a link-tracing process that aimed to identify chains for actors involved in the supply of chickens from farms to consumers and agreed to the recurrent presence of two Chennai-based researchers in their retail outlets to understand and document their operations in detail. Building on these initial and in-depth insights into downstream retail enterprises, we relied on link-tracing and snowballing from interlocutors to recruit broiler wholesalers, following leads and contacts as they emerged through the research process.

Further contacts were provided by veterinarians and academic staff at the Tamil Nadu Veterinary and Animal Sciences University with knowledge about and contacts in the industry. We also soon learnt about the Chennai Poultry Wholesaler Association (CPWA), which established wholesalers that we initially interacted with are members of, and through which we obtained further contacts especially to association members who had a significant role in it currently or historically. Not all wholesalers with whom we spoke were CPWA members, however, and we actively attempted to learn about and seek contacts to wholesale trading actors that pursued different business models and trading practices. We thus ended up speaking to a range of different wholesalers, from traders who procure from farms—which were the initially intended focus—to wholesale intermediaries operating within Chennai. For example, we spoke to traders who understood themselves as primarily wholesale actors procuring from farms for wholesale supply to Chennai, and others who started as retailers and entered wholesale procurement to increase their margins. While our study was not designed to be statistically representative of all business models and trading practices, the length and depth of our engagement with the sector provide confidence that we captured a comprehensive understanding of common trading modalities and practices. We did ask traders about their trade volumes, and extrapolating these aligns with orders of magnitude that we would expect for broiler supply into Chennai as mostly fulfilled by CPWA members. While large established traders reported turnover volumes consistent with an oligopolistic market structure in supplying Chennai, for example, several 100 tonnes per month, we also spoke to smaller traders who reported as little as 6 tonnes per month. However, as reported volumes may be subject to under- or overestimation and could not be independently verified, we chose not to provide numbers for indicated volume and did not stratify traders by size.

Interviews were primarily conducted with enterprise owners or general managers and sometimes complemented by interviews with others such as branch managers and retail staff. Two wholesaler enterprises and their branches were the initial focus. Research processes with them involved repeat visits and interviews with actors across their operations. Research with subsequent wholesalers became increasingly targeted to management levels and were eventually limited to single interviews or interactions for complementary perspectives. In total, 30 interviews were conducted with interlocutors from 13 wholesalers in Chennai. One further broiler wholesaler in Namakkal was visited for a complementary perspective, and some other retail enterprises in Chennai to corroborate elements of their business relationships with wholesalers and to obtain wholesaler contacts. Two broiler integrators, including a hatchery and a contracted farm, were also visited to gain a better understanding of the production side for context.

We initially formulated some guiding questions surrounding the operation of broiler retailers and wholesalers. Following the initial phase of familiarising with retailer operations, we formulated more specific and extensive interview guides for the research process with broiler wholesalers. These broadly covered questions about their background and details of their role, relationships up and down the PDN, financial matters and transactions, relations with other relevant actors and their knowledge about them, biosecurity practices and any challenges they experience in their businesses. These served as a guide for initial structuring of interviews and research interactions, with the research process being adapted in accordance with the dynamics of interaction with interlocutors and emergent findings. We had intended to also gain a better understanding of profit margins in broiler trade, but it became clear quickly that this would have required a different methodology to generate reliable data [34].

Most interviews were audio recorded following informed consent of interlocutors after explaining the purpose of the study and handling of information. In some instances, interlocutors declined to have interviews recorded but agreed for researchers to take notes. Generally, interviews were conducted in Tamil by two Chennai-based researchers (GJ and VP) who were closely supported in study design and methodological approach by two UK-based researchers (IS and EH) and two Delhi-based researchers (PM and KY). The Chennai-based researchers combine backgrounds in biological (GJ) and social sciences (VP) and partners in India and the UK contributed further qualitative social science expertise through IS and EH as anthropologists and PM and KY with expertise in international politics and global and public health. A few field visits and interviews were joined by a UK-based researcher (IS) with rudimentary Tamil knowledge. Interactions and interviews during these field visits were conducted in Tamil and English with translation by the Chennai-based researchers where required. Field notes on observations and interactions in retail and trading enterprise premises were taken during visits and complemented afterwards. In line with participant observation in ethnographic approaches, field notes tended to be broad, capturing general details and subjective impressions about spaces, what happened in them, and the interactions researchers had. Rather than generating systematic observational data across visited premises that could be numerically evaluated, such field notes served to contextualise the understanding of business models and practices, assist their interpretation and bring up questions to follow-up on in subsequent interactions.

Despite our confidence about the insights gained in this study, some obvious limitations remain. Most importantly, reliance on trader narratives may underreport risky practices. This holds especially for practices related to procurement from farms, which was also beyond the scope of empirical evidence through observation. Such limitations also relate to responses to clinical signs in birds. All traders brushed off questions about disease by stating that they only trade healthy birds of the highest quality. While this may be their core business model and reputation that their trade relies on, it is not credible to assume that visibly sick birds would be entirely absent in such broiler trade. A gap in this study thus remains the understanding of informal trade segments with, or the disposal of, lower quality or potentially diseased birds. A further limitation is the lack of triangulation of information with producers that wholesalers procure from, such as integrators and farmers. These are limitations that future studies should consider and address. Finally, the focus of this study has been the understanding of business models and practices in broiler wholesale trade and the configuration of value chains. While the insights of this study point to some specific recommendations, from a social science perspective we would urge caution in translating these findings into blueprints of interventions towards best practice for reducing risk. Identifying interventions to reduce risk should rather be done through further work so to co-develop solutions that are practical and achievable in their context.

Ethics approval has been obtained from the Social Science Research Ethical Review Board at the Royal Veterinary College in the UK (reference URN SR2020−0265) and by the Institutional Ethics Review Board, Jawaharlal Nehru University, in India (reference 2019/Faculty/185).

### 2.2. Data Analysis

Interviews and notes from interactions and observations were transcribed and where applicable translated into English by GJ and VP. Transcriptions took place concurrently with the research process and were checked by at least one UK-based researcher. Initial transcriptions were read by two UK-based and two Delhi-based researchers to guide and adapt the research process in accordance with initial findings. All data were entered into MAXQDA for coding and analysis. Coding was conducted deductively based on the research objectives and inductively following emerging themes in the data. After initial coding of most data, codes and code clusters were consolidated in a workshop in Chennai with GJ, VP and IS. This led to some reorganisation, merging and differentiation of codes. Remaining data was then coded, and previously coded data cross-checked in relation to revised coding and any potentially missed information. Generally, coding and analysis followed an iterative process of engagement with the data, congruent with ethnographic approaches to analysis and writing [33].

## 3. Results and Discussion

### 3.1. A Time When Traders Ruled the Market

To understand today's shape of the broiler PDN and the role of wholesale traders, we need to place current wholesale operations into historical context. The early days of the broiler market in Tamil Nadu were very different from the current PDN. Although imported fast-growing broilers were introduced to India in the 1960 and 70s [35], it took a while for broilers to become a popular commodity. One trader long in business recalled how they were trading ducks and duck eggs in the early 1970s and then gradually incorporated chicken into their trading. This started with native chickens and eggs. They suggest there was high demand for the relatively little supply of poultry at the time and that their procurement range could thus span considerable distances. Poultry markets in Chennai, especially in Pudhupettai area, were hubs of live poultry trading, in particular ducks, back then. Most poultry—reaching Chennai in bamboo baskets transported on buses and trains—transited through these markets. Broilers were brought to them as a new product.

Although both high-yielding broiler and layer had been introduced to India by then, it was the layer breeds that initially became a success. One trader called the cockerels of the Babcock layer breed ‘the king of chicken' during that time. Broilers became increasingly available in Chennai during the 1980s, according to another trader, and steadily increased in significance until they became established as the dominant live poultry commodity in the 1990s.

With growing demand for chicken meat in the 1970 and 80s, chickens were increasingly produced in Chennai's closer surroundings. As broilers became more popular, people are said to have been rearing them even within Chennai on the roofs of their buildings. Into the 1990s, production in Chennai and its suburbs is said to have increased to extents that satisfied a good proportion of the city's demand for chicken. Most broiler production back then was still conducted independently by individual farmers. Hatcheries sold day-old chicks to farmers, and other companies started specialising in selling premixed feed.

This geography of production and procurement for the Chennai market changed during the 1990s with the adoption of stacking cages of chickens on trucks enabling the mass transport of broilers—for example from Tamil Nadu's western districts around Coimbatore where Suguna started broiler contract farming in the early 1990s—to major cities including Chennai. Companies such as Suguna are also colloquially referred to as ‘integrators' in the Indian poultry sector, referring to the vertical integration of chicken PDNs through contract farming schemes, supplying day-old chicks and feed to farmers who raise them for a commission [36–39].

Contract farming grew rapidly since the 1990s and it can be assumed that the ease of mass transport of broilers helped its growth towards the predominance it has today. The FAO reported industry sources suggesting that 90% of broiler production in Tamil Nadu was under contract by 2005 [40]. This transformation of the sector has been referred to as India's poultry revolution [35]. Integrators are now active across Tamil Nadu and beyond—both large established and small companies—and gradual shifts in production hotspots are taking place from major integrators' initial production areas to other places with suitable climate and proximity to downstream markets. In Tamil Nadu, this means increased production in areas such as Krishnagiri, located between the major South Indian cities of Bengaluru and Chennai. At the same time, real estate investment and speculation pushed agriculture and livestock production further away from Chennai's immediate surroundings [41].

Looking back, traders reflected on the considerable market power they enjoyed into the 1990s. As one of our interlocutors stated:

*‘People used to rear chicks; they raise them and sell them. That time, marketing co-ordination was not there, many of them don't know the price. If a trader goes to procure chicken, he is the only authority to fix the price'*.

Another trader characterised their business then similarly as:

*‘During those days only traders decided the rate; the trader was a powerful person'*.

Clearly, traders held the predominant role as market setters at the time. This is in line with research conducted on the governance of different agri-value chains in India, including poultry, that characterises them as relatively informal markets that were driven by traders [42], and corresponds to what Gereffi [24] refers to as ‘buyer-driven' value chain. It is also congruent with research on agricultural markets in South India, and Tamil Nadu in particular, conducted in the late 1970s and 1980s, which saw a shift in terms of what had been dubbed the ‘masters of the countryside' as being no longer large local landowners but rather an urban ‘mercantile class deriving most of their resources and economic power from marketing' [43]. The prominent role of wholesale traders has also been documented for other agricultural commodities in South Asia, such as rice in Bangladesh [44].

Chennai's poultry traders, initially concentrated in the Pudhupettai area, expanded into other locations of the city with the increased supply of broilers in the 1990s, enlarging the reach of their business through additional wholesale supply points. Others also entered the Chennai-based wholesale broiler trade at the time, usually as a change of role in the poultry sector as a veterinarian or prior employee in a poultry company. Wholesalers then also invested in fleets of large commercial vehicles to procure broilers, corresponding to the changes in production areas away from Chennai and the ease of mass transportation. Throughout these changes of the sector, Chennai wholesale traders became more specialised. Most traders we spoke to focus on broiler chickens exclusively rather than trading in different kinds of poultry, while having expanded their business operations at the same time. This resulted in a relatively small number of consolidated trader enterprises dominating the Chennai wholesale broiler market. At the same time, the rise of the poultry integrators and thus a more organised production sector meant that traders lost their position as sole market setters. The shift to integrator dominance reflects a ‘producer-driven' chain [24], where integrators control production inputs (day-old chicks, feed) and dictate farmgate prices. This contrasts with Chennai's earlier ‘trader-driven' phase, where wholesalers exerted pricing power. As one trader characterised summarily:

*‘First traders controlled the market but now the control is in the hands of the producers'*.

Such historical shifts in the configuration and governance of PDNs have implications for how biosecurity is managed and incentivised along them. Vertical integration, for example, can provide for centralised biosecurity oversight [22]. Yet, vertical integration may also generate the kinds of scales and spatial relations of production, trade and connectivity within and across value chain segments that heighten the risks of disease emergence and transmission, making biosecurity such an important concern in response to pathological possibilities in the first place [45, 46]. As Rich and Perry [25] point out, distributional issues and power relationships among PDN actors can play a large part in determining the incentives and economic trade-offs of biosecurity investments along the chain.

### 3.2. The Configuration of Chennai's Live Broiler Market Today

While some large integrators also have processing facilities and market dressed chicken products via supermarkets, relying on cold chain infrastructure, the live broiler trade that we focus on here remains the most prominent channel for supplying Chennai with chicken meat. At the most basic configuration of this PDN, Chennai wholesalers procure broilers from the established integrators. Wholesalers then distribute live broilers to retailers and potentially other actors engaging in intermediate wholesale. Wholesalers engage in retail themselves to varying extent and some also supply dressed chicken meat to wholesale clients such as restaurants, canteens, caterers or other intermediate suppliers. Beyond simply constituting the network link between production and downstream destinations, wholesalers pursue different business strategies to capture value from the PDN.

Today, the wholesale live broiler market in Chennai—as mostly in the hands of a relatively small number of entrepreneurs or family enterprises—is perhaps at its peak of consolidation in its current configuration. According to several of our interlocutors, there are about 40 broiler wholesalers organised in the CPWA. Not all broiler wholesalers are members of the association, but most of the large established traders apparently are. The association functions as a coordinating body among members but also potentially as an organised group to lobby or negotiate with other actors such as integrators or the state government. For example, it sets daily wholesale broiler prices for downstream sales in Chennai as coordinated among its members, a guiding maximum price, which are published in newspapers and their freely accessible mobile app.

Thus, while the established Chennai-based broiler wholesalers are competitors, they are also a group of entrepreneurs with common interests who seem to work more in amicable coordination than antagonistic competition. For example, several wholesalers mentioned that they have their geographical reach within Chennai and avoid territorial competition with other wholesalers. As one wholesaler framed it:

*‘There will be a trader in all the areas of Chennai. So, we have some distribution radius. We won't disturb other traders'*.

Although wholesalers are not in control of market prices along the PDN like they once were, a relatively small group of consolidated businesses has, over the past decades, come to exert a certain oligopolistic control over Chennai's broiler market. From a biosecurity perspective, this trader-led oligopoly exemplified by CPWA's price coordination appears to prioritise market stability over collective biosecurity investment. This could be considered as a trade-off between market concentration and biosecurity [25]. The horizontal governance structure of mutual coordination, for example, may fragment accountability. While the territoriality among wholesalers likely limits pathogen mixing, it may also discourage shared risk mitigation.

### 3.3. Upstream: Procurement and Production

Wholesalers procure broilers from farms associated with integrators. A wholesaler tends to work with a limited number, for example, two to five, of the large integrators with whom they maintain regular business. None of them maintained contact with individual independent farms, meaning farmers who produce broilers outside contract farming arrangements. Wholesalers make decisions about procurement locations considering the availability of broilers with integrators—or rather their regional branches—and daily fluctuations of farmgate broiler prices between different areas. Integrators determine their farmgate prices based on maximum prices set by Broiler Coordination Committees (BCCs), constituted by representatives of the major integrators. BCCs set prices for areas typically covering one or several districts, although not necessarily aligning with administrative district boundaries. They are generally relating to the Vencobb breed—an adaptation of a Cobb breed to Indian conditions by Venkateshwara Hatcheries, known for their poultry brand Venky's—as the broiler breed with the highest market share, which wholesalers estimated at around 78%–90% in production areas relevant to them. While not binding for producers of other breeds, the price of Vencobb broilers has broader guiding effect for farmgate broiler prices generally.

The procurement areas appear similar for Chennai-based wholesalers, both as there is a limited number of major integrators and as these major integrators tend to be widely present and thus overlap in production areas. Some variation can be expected based on the specific integrators that a wholesaler works with. It is difficult to determine the exact number of integrators supplying Chennai, but there are about 10 large integrators frequently named among wholesalers. One integrator we spoke to suggested there might be about 50 integrators of varying size active in Tamil Nadu. Not all of them, however, operate at a scale and in areas that make them relevant for Chennai wholesalers who work with large integrators that can guarantee uninterrupted supply in relative proximity to Chennai, usually corresponding to northern Tamil Nadu and southern Andhra Pradesh.

Besides farmgate prices, wholesalers consider the distance from Chennai as affecting fuel and staff costs, chicken weight and welfare. To prevent heat stress, broilers are procured overnight. During the hotter months, transport volumes are reduced to allow increased airflow between cages. Wholesalers generally experience some mortality among chickens during transport and observe some weight loss between weighing broilers on farms and then again for onward sales in Chennai. Mortality during transport can also be caused by injury while ‘loadmen' fetch them on farms and place them in cages.

For procurement, the large wholesalers use trucks with a capacity for carrying about 5 tonnes of live birds. While numbers of how many such trucks for procurement wholesalers own vary, none claimed owning more than 10. Wholesalers usually procure chicken daily but higher volumes for Sunday, which is the day of highest demand, and Wednesday, also a day of higher demand. In times of high demand, some complement their procurement by hiring additional transport. There are some transport agencies specialised in broiler transport, and other wholesalers based in production areas or only acting as bulk wholesalers who deliver to Chennai-based wholesalers. The overall highest trade volumes traders we spoke to mentioned were around 600 tonnes or 300,000 chickens per month.

Some wholesalers have also started to expand upstream with producing broilers themselves. These are significant steps of business expansion that require substantial amounts of human resources and capital investment. While a strategic choice for some wholesalers, presumably not all wholesalers would have the financial capacity to engage in such step. In effect, some wholesalers became integrators themselves, contracting farmers to produce broilers. They supply farmers with day-old chicks, feed, production supervision and ultimately pay them a fee for the ready birds. One wholesaler we spoke to had recently started their integration venture and was going to upscale from the initial 50 farms they started working with. While operations vary in scale, in rough order of magnitude wholesalers with established contract farming schemes may work with hundreds of farmers in a specific area, whereas the larger integrators work with thousands of farmers across different areas. This upstream expansion into production is a strategy by some wholesalers to capture more value from the PDN. As such, it is also a reaction to the increased competitiveness of wholesale and retail marketing in Chennai, which limits the volumes and margins to be made in the broiler trade. Wholesalers who expanded upstream may still complement their broiler procurement through other integrators rather than entirely relying on their own production through contract farming.

Regardless of whether wholesalers procure broilers from other integrators, their own contracted farms, or a combination of both, in rare instances some wholesalers may also approach other Chennai-based wholesalers to make up for an unforeseen higher demand than their procurement on the day. This is not a regular procurement strategy for established wholesale enterprises, but rather a response to fill an unexpected shortage at short notice. As one wholesaler put it:

*‘Usually we do not procure from any other trader. When we send our vehicles for procurement, we procure surplus quantities. We always procure 1500–2000 kg extra to avoid crises. Mostly we don't ask other traders, rarely once in 6 months due to transport issues [like a vehicle breakdown or accident] we may buy from other traders. That may happen sometimes unfortunately, we can't avoid that kind of incident. That time we are looking to traders'*.

Further, wholesalers procure broilers based on their clients' demands. For example, wholesalers supplying restaurants require birds of distinct weight specifications than the birds usually procured for retail. While the regular preferred procurement weight of a matured broiler chicken for retail is around 2 kg, restaurants demand smaller chickens of around 1.6 kg live weight for tandoori and grilled chicken dishes and larger birds of 2.5 kg or more for boneless chicken dishes. Depending on respective procurement volumes, this may involve procuring broilers of different sizes, and thus from different farms, through a single truck. For example, a single vehicle may procure some smaller birds from one farm and fill remaining transport capacity by procuring regular weight broilers for retail from another farm. When higher numbers of smaller birds are required, it likely involves more farm visits, as these are collected by thinning flocks rather than integrators selling entire flocks prematurely. In general, wholesalers prefer to visit fewer farms for logistical ease, so may consider going longer distance if that facilitates collecting from a single or fewer farms. Some wholesalers indicated that of three trucks sent out for procurement, on average, two go to a single farm and one goes to two different farms for collection. It appears rare that a truck collects from more than two farms, but further research will allow corroboration of this.

In relation to mature broilers, a wholesaler who also expanded into production mentioned that from the perspective of an integrator, slightly older and heavier broilers would optimise their gain. Wholesalers, however, prefer relatively younger birds with more tender meat, for example a live weight around 2 kg and 35 days old, as these corresponded to consumers' and, therefore, retailers' preferences. Similarly, from the perspective of optimal meat yield from a carcase, a preference for Ross breed chickens (marketed by Aviagen, a subsidiary of EWG) was mentioned. At the same time, it was acknowledged that the Ross breed is more delicate and prone to disease and mortality and thus less profitable to raise in the warm South Indian conditions, driving up production costs, and that thus most integrators nowadays work with the Vencobb breed. Two major integrators, SKM and IB Group, continue to produce Ross chickens within procurement radius of Chennai while Suguna is said to have discontinued production with this breed.

Major integrators have in the past used different ways to promote themselves among Chennai-based wholesalers. Generally, it is the wholesalers that major integrators seek to cultivate as clients, while retailers request broilers of certain weight specifications without necessarily knowing through which integrators wholesalers source them, even though butchers at retail outlets were able to point to differences in breeds. Wholesaler offices for example display trophies and awards they were given as part of promotional activities by integrators. They also reported that in the early 2000s integrators took them on foreign trips, for example to Malaysia, Singapore or China. For retail outlets, integrators provided—and some of them still do—name boards that prominently display the integrator's poultry brand name with the shop name printed at the bottom. This not only serves to popularise their brands among consumers. When asked whether the integrator provided any money or other benefit in return for having their branded name board installed, one wholesaler for example recounted how the display of the company's brand on their retail shop's name board created a kind of identity through the apparent association with a well-known corporate brand. As he put it:

*‘Once, I felt ashamed to mention my profession during my daughter's school parents and teachers meeting but today I am saying this to everybody. They gave me this identity'*.

### 3.4. Marketing of Broilers: Core Business With Different Destinations

**Figure 1:**
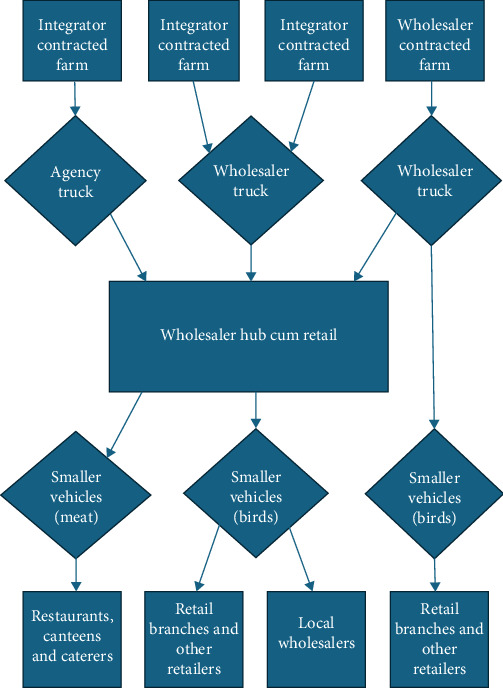
Schematic overview of broiler bird and meat trade in Chennai from a wholesaler-centric perspective. The figure intends to illustrate different nodes and potential pathways in terms of upstream procurement and downstream marketing rather than reflecting exhaustive value chain configurations. Arrow between wholesaler truck and smaller vehicles reflects shifting birds directly between vehicles rather than transiting them through wholesaler hubs.

Chennai-based wholesalers share the role of procuring broilers from production areas for marketing them in Chennai. However, there are different market segments and strategies wholesalers engage in to varying extent. One common market segment is to supply their own and other retail outlets. Many wholesalers may thus be better characterised as wholesaler-cum-retailers. However, the extent to which wholesalers engage in retail themselves varies, and most retail shops in Chennai are run by independent retailers. Further, there are a range of wholesale marketing destinations that entail different kinds of market relations and transactions, such as restaurants and canteens of both private and public sectors, and other wholesale intermediaries that supply retail and food catering enterprises. A schematic overview of procurement and marketing pathways from a wholesaler perspective is presented in Figure 1.

Wholesalers maintaining retail outlets themselves is reflecting, on one hand, the historical configuration of broiler PDNs in Chennai, and, on the other hand, an attempt to maximise value extraction along the PDN by retaining the margins of retail sales within the business. Some wholesalers thus explicitly focus on own retail operations in terms of marketing and place less emphasis on wholesale supply to other actors. Some also started as retailers, and procuring directly from integrators was a way to increase margins through also assuming the role of wholesale traders that they previously relied on. Presumably few traders, however, if any, maintain an exclusive wholesale procurement for own retail without any wholesale supply to others in some form.

Several of the wholesaler-cum-retailers, however, mentioned increasing challenges in the retail sector due to growing competition by new entrants. While previously a strategically placed retail branch would be the go-to shop for an entire neighbourhood, nowadays an increasing number of retail shops compete for the same customer base. The established wholesaler-cum-retailers, thus, report losing some of their traditional retail market share, decreasing turnover per branch. Given the overhead costs that each branch requires, some wholesalers reported closing retail branches as they become less profitable. One trader mentioned that they reduced their retail branch network from 32 to less than 10. While retail marketing remains an important part for many broiler traders, there is a general sentiment that retail is becoming more challenging due to increased competition.

At the same time, increasing numbers of retail operators widen the potential client base of wholesalers. For many wholesalers this constitutes the core element of their business. Although in this segment competition is also reportedly increasing. This manifests, for example, when regular retailer clients order more intermittently from their usual wholesale suppliers. This indicates that retailers are increasingly looking around for competitive rates rather than staying with their regular supplier. Wholesalers, thus, also change the way they transact with retailers. While it seems common to have retailers pay wholesalers weekly or even fortnightly for the supply they received over the period, the changing business environment makes wholesalers increasingly take payment from retailers directly upon delivery.

Wholesalers observe a lack of loyalty not only by their clients, but also by their own employees. The latter may set up their own wholesale businesses and take a part of their wholesaler customer base with them. It is such new entrants into the wholesale segment that offer more competitive rates to retailers, rather than competing established wholesalers who rather coordinate their pricing and distribution radius. Established wholesalers suggest that many of these recent competitors often disappear as fast as they emerge, as they allegedly try to cut costs through improvised business setups that are ultimately not sustainable, for example, operating with improperly maintained vehicles and ceasing business when vehicles break down. From our interaction with retailers, it also seems common for them to maintain regular business relations with two wholesalers, thus reducing supply risks inherent to relying on a single source.

Besides the live bird trade, there is a range of client types for dressed chicken orders with different respective pricing and transaction regimes. This starts with small local restaurants and their comparatively low quantity requirements. These may purchase and collect directly from wholesaler branches and tend to be charged retail prices like other walk-in customers. They pay immediately in what wholesalers refer to as ‘cash and carry'. Larger restaurants with regular demand of higher quantities tend to be charged wholesale live bird rates plus an additional 10 or 12 rupees per kg for slaughter, get their orders delivered daily by wholesalers and may bundle payments following several deliveries. These may not be clear-cut categories and specific arrangements vary based on specific personal and business relations.

High-class restaurants, hotels and organisations running canteens such as colleges, hospitals, or corporate clients such as IT companies often seek longer-term supply agreements with fixed rather than daily market rates and monthly payments. Sometimes wholesalers have agreements directly with these clients, for example, restaurants, but often their agreements are with intermediaries contracted to supply caterers or canteens. These are supply conditions only large wholesalers can fulfil. It requires considerable capital to deliver several 100 kg, or more, of dressed chicken with payment only forthcoming following a month of supply or even longer. These arrangements also carry specific risks. Wholesalers face difficulty recovering their dues if such clients go out of business. Market fluctuations in broiler prices can also eat into margins when fixed rates for supply have been agreed. Some wholesalers were explicit that they decided not to engage in this kind of trade and instead focus on wholesale supply of retailers and their own retail sales that they regard as more secure in terms of regular payments and control over margins according to daily price fluctuations. Smaller traders may stay away altogether from regular supply arrangements with restaurants that involve any payment delay. As one smaller trader put it:

*‘I don't supply to restaurants, money will be stuck as they will settle the amount late […] I will supply only for retail shops […] This is going smoothly, but restaurant people will settle the amount 2 days later. In between, another 2 days payment will be pending. Like this I have faced much loss in restaurant supply'*.

Two of the wholesalers interviewed also turned out to be marketing agents or acting as middlemen for other traders. They do not procure broilers directly from integrators, but act as agents connecting a wholesaler procuring from production areas to downstream wholesale clients for a commission or agreed portion of the selling price. They do not own vehicles, although they may have a retail shop, and may also purchase and stock broilers in bulk themselves to resell to wholesale clients from their own storage space. In practice, they communicate volumes required by downstream clients and themselves to wholesalers. Wholesalers may then deliver to downstream clients directly and the additional stock requested to the wholesale agent's retail or storage space. One of these agents interviewed, for example, maintains a farm-like setup on the edges of Chennai where he indicated to keep broilers for up to 5 days for collection by nearby retail shops, for example, through hired small tricycle transporters. One of them detailed that they receive seven rupees commission per kilogram from the wholesaler and that they pass on a discount of three to four rupees to their clients. By sharing their commission with their own wholesale clients, they can sell broilers at competitive rates. The other wholesaler of this kind mentioned a similar arrangement of receiving a commission of eight to 10 rupees and passing on a discount of maximum four rupees to their clients.

Others still, although more of a niche, increase their value extraction from the broiler PDN through business with by-products. One wholesaler, for example, made a name for themselves by collecting slaughter waste across Chennai, which they process in a rendering plant and market as pet food. In other instances, while less organised, slaughter waste is collected to be used and sold for fish farming. Wholesalers and retailers are happy to have the slaughter waste collected by actors who have an interest in it, as it is otherwise a burden to dispose of, which municipal waste collection services are also hardly able to keep up with. Wholesalers reported that previously a lot of slaughter waste would have been left on roadsides but that this is less of a problem nowadays. All these destinations pose their own epidemiological risks, albeit different ones, from the environmental contamination of slaughter waste on roadsides, which may also get picked up by wild birds and stray animals, to the potential of introducing pathogens to aquaculture systems by using slaughter waste in fish farming. The practice of using leftover products from poultry slaughter in fish farming has also been reported from Bangladesh, where the frequent marketing of chickens without adherence to antimicrobial withdrawal periods raises concerns about the potential creation of reservoirs for antimicrobial resistances [47].

### 3.5. Epidemiologically Relevant Management of Wholesale Hubs and Transport

Towards assessing the epidemiological significance resulting from the network configuration presented above, and specifically as relating to the way wholesale hubs where birds are stored and the transport of live broilers are managed, we here explore factors that influence: (1) the mixing of birds from different species and origins, and of their pathogens, (2) the amplification of pathogen circulation along the PDN and (3) the spread of pathogens from wholesale hubs or retail outlets to farms.

Broilers that are not delivered to retailers are kept at wholesalers' trading hubs. This may involve mixing of broilers from different trucks and origins and thus, potentially, their pathogens. At the level of a wholesaler, relevant factors are the collection of chickens from different farms through the same trip, and the subsequent mixing of birds from different procurement trips in wholesalers' trading hubs. While we do not have quantified data about the number of procurement trips and farms from which broilers are mixing at wholesale hubs, these are significant arenas for the potential of pathogens to transmit among birds of different origins. The collection of birds from multiple farms by a single truck also creates risks for pathogens to be carried and introduced between farms. At the level of Chennai's live broiler trading network in its entirety, different factors are at play. For example, the territoriality of areas that Chennai's established wholesalers tend to supply and the absence of regular trade among them limits the possibilities of mixing broilers and their pathogens across Chennai's trade network. While quantitative network analyses would help to corroborate its extent, this appears to differ, for example, from the trading practices and strong epidemiological connexions documented across major urban trade networks in Bangladesh, where within-market viral genetic diversity is almost as high as among all markets within a given city [48, 13]. Trade networks of wholesalers are not entirely distinct, however, as wholesalers procure from an overlapping network of integrators, their contracted farms, and geographical areas. Territoriality among wholesalers may thus reduce connectivity compared to Bangladesh's dense trade networks [13], potentially limiting pathogen mixing and diversity in retail outlets. However, procurement from overlapping integrators creates indirect linkages, enabling pathogen circulation across otherwise separate trade networks. These overlaps might be less where wholesalers have furthered the vertical integration of their trade network from own contract farming production to retail. Independent retailers tend to regularly work with more than one wholesale supplier to mitigate supply risks, thus in principle also increasing risks of pathogen transmission between birds of different origins.

Besides the potential for birds from different origins and therefore their pathogens to mix, there are factors that may promote the transmission of pathogens among chickens along the PDN and therefore the amplification of their circulation from production sites to retail shops. These relate to the density and length of time that chickens are kept in trading hubs, which could thus become pathogen reservoirs [49]. All wholesale hubs and retail outlets have a space where broilers are kept after their removal from cages. The storage capacities wholesalers mentioned for their hubs and retail branches range from 100 to 2500 birds. One wholesaler specified that in trading they keep broilers more densely than is usual in farming, with up to one broiler per 0.75 square feet, thus keeping up to about 2500 birds in a storage area of 2000 square feet. As wholesalers tend to procure some excess to the expected demand, there is a good chance of leftover birds at the end of the day. Any leftover broilers tend to be sold first the following day (d + 1), and according to wholesalers it is rare that broilers stay with them till another day (d + 2) and never longer than that. An exception to this was the intermediary wholesale agent introduced above who keeps broilers in a farm-like setup for upto 5 days. When left from previous days, mixing with birds from new procurement may occur, although we also observed improvised barriers between broilers from different days in storage spaces. At retailers, broilers may also be kept in relative proximity to other poultry, such as native chickens or quails, but usually not together. Although many retailers use cages to display different types of poultry in front of their shops, these may be stacked above each other. These practices allow for potential pathogen transmission between poultry of different provenance and production systems. Further, heat stress during transport may increase chickens' susceptibility to pathogens and enhance pathogen transmission [50].

Further, there are factors that may influence the spread of pathogens from wholesale hubs and retail outlets back upstream to farms, for example, through vehicles and the cages used for transport. Cages circulate from farms via wholesale hubs and retailers back to farms. For example, when cages with broilers are brought into Chennai, they are distributed to smaller vehicles and then delivered to retail outlets. They often stay at retail outlets until being collected later in the day again, so to be used in the next procurement trip from farms. Cages can thus potentially act as fomites, spreading pathogens from broilers passing through wholesale and retail back to farms. Wholesalers have also been consistent in their accounts about cleaning vehicles and cages. The cleaning regime is usually limited to roughly sweeping off any dried accumulated material on vehicles such as chicken faeces. Wholesalers we spoke to generally did not seem to perceive any pathogen transmission risks to emanate from transport, or for that to be relevant for broilers on the way to be slaughtered and, thus do not associate any specific cleaning regime with the health and quality of mature broilers procured from farms. Usually, no wet cleaning or application of detergent or disinfection agents takes place. These practices are unlikely to be sufficient for preventing cages and vehicles to act as fomites between sites, as improper cage cleaning likely facilitates fomite transmission.

This cleaning regime is similar for the spaces in wholesale hubs and retail outlets where broilers are kept, usually in a separate area with a concrete floor. This presumably has to do with the dry and hot climate throughout most of the year. One retailer, for example, was explicit in his objection to wet cleaning the areas where chickens are kept. According to them, the evaporating water would generate humidity and increase the sense of heat, affecting chicken welfare and appearing to create more muck than brooming away the quickly dried faeces, and thus potentially pose higher risks to chicken health than potential disease spread. Wholesale and retail spaces through which cages pass may thus also become reservoirs for pathogens.

An exception to the cleaning regime of vehicles was mentioned by a wholesaler who also engages in broiler production through their own contract farming. They do follow a different cleaning regime when deploying their vehicles to deliver day-old chicks from the hatchery to their contracted farmers. For this, trucks are cleaned and disinfected more thoroughly, to prevent any risks that residues from broiler transport could pose for the health of the chicks. This was explained with the impact that disease could have for the growth of chicks, whereas mature broilers are on their way to be slaughtered and, thus the potential of pathogen transmission is not a concern in the same way.

### 3.6. Dynamics of Change in Chennai's Wholesale Trading

While wholesale broiler traders organised in the CPWA will remain the principal actors and beneficiaries of the broiler trade into Chennai for the foreseeable future—barring more radical changes—we see three main tendencies that are likely to affect the future configuration of Chennai's broiler market. First, the increasing presence of corporate actors in Chennai's poultry meat market through branded retail outlets for the marketing of meat from live broilers, on one hand, and through the attempt to shift increasing market shares to the chilled and frozen meat market based on centralised and industrialised slaughter facilities and cold chain infrastructure, on the other hand. Corporate shifts toward chilled meat mirror global trends toward financialized agri-food systems [51, 52]. This may exclude small actors, pushing them into riskier informal trade [53]. Second, the drive to further vertical integration upstream by Chennai-based wholesalers, expanding their own production through contract farming. And third, a further multiplication of actors in wholesale and downstream intermediary and retail roles, chasing small margins through improvised and often informal enterprise models in an increasingly competitive wholesale and retail market. These tendencies, described in more detail below, will likely continue in parallel in the foreseeable future, and the assessment and mitigation of epidemiological risks emanating from live broiler PDNs in South India will have to pay attention to the resulting diversity of trade network configurations and associated risks.

Above we suggested that the consolidation of Chennai wholesale businesses in their current form might be at their peak now. Why do we say so and how are wholesalers experiencing the dynamics in Chennai's live broiler market? According to wholesalers, the strong demand in broilers and its projected further growth led to an increase in production that came to outstrip demand in recent years, leading to price fluctuations and more intense pricing competition. This provides a challenge for wholesalers whose margins thus decrease. As one wholesaler put it:

*‘As a whole, traders are still comfortable, it's not a big deal, but the thing is, again, among traders there has been a lot of competition over the years, even though there is an increase in sales, competition has also outgrown it, so traders, like, it's not as comfortable as it was before, 2015 or 2010, the market was much more… Look, as with any product, if the demand is higher everyone makes money in the chain, it's a pipeline, but when the demand drops and the production is more, then someone will have to lose out something in the pipeline'*.

The dynamics of increased competition seem to have been further catalysed through the COVID-19 pandemic. For example, as a wide range of economic activity was restricted, poultry trade continued to be regarded as essential and legal to pursue when a lot of other trade and economic activity became disrupted. This saw new actors—who lost their usual source of income—entering the poultry trade. On one hand, this produced new actors engaging in mobile or roadside sales. This assisted wholesalers' turnover during times when they faced limitations in operating their retail outlets—both in relation to permitted opening hours and customers willingness or ability to travel to and enter shops. However, on the other hand, it also increased the entry of more short-lived and less organised traders attempting to capture some margins through poultry wholesale. The pandemic-driven informalisation also aligns with Indrawan et al. [22] findings that economic precarity forces actors into high-risk, low-margin trade. Subsidies for hygienic slaughter (e.g., community rendering plants) could mitigate risks without displacing livelihoods.

In the words of our interlocutors, the pandemic further changed the perception of poultry trade as an economic activity. Previously regarded as ‘dirty' and beyond many entrepreneurial market-seekers' imagination, the pandemic shifted the perception towards poultry as being a lucrative business, shedding its connotation of a ‘dirty' niche. The connotation of ‘dirty' is deliberately in quotation marks as it seems to speak to a sociocultural shift in symbolic perception. Dirtiness is not necessarily an opposition to cleanliness here, in terms of the appearance of a chicken retail shop for example, but rather as standing in contrast to the values of purity associated with the vegetarianism of higher caste Hindus. As Bruckert [32] discusses, the perceived dirtiness and impurity of chickens that underlined its historically low consumption in South India—as consumption of meat in general but also specifically in comparison to mutton as meat from an animal with perceived traditionally less ‘dirty' eating habits—has given way to chicken meat now being perceived as the most acceptable and in fact most consumed one. The pandemic, pushing a variety of people who previously earned their living in other ways that came under pandemic restriction to explore working in chicken trade and retail, seems to have furthered this shift in perception. Nevertheless, the perception and stigmatisation of chicken trade as dirty probably also underscores a political economy of neglect, in which actors lack the capital to modernise and thus perpetually operate at the margins of high-risk informality, aligning with Rich and Perry's [25] findings on underinvestment in biosecurity among marginalised actors.

The increasing presence of new entrants into Chennai's broiler wholesale by actors that seek to compete with the established wholesalers—and outside their informal territorial coordination—may add to the connectivity of trading networks through the multiplication of actors competing for ever smaller margins. Even if many of these competitors are said to not stay in the market long, seeking quick profits with little capital and without factoring in the costs and risks of the wholesale broiler trade such as price fluctuations and accidents, the growth of a more unstable and precarious informal segment of wholesale and downstream segments may bring its own dynamics for the potential of pathogen transmission through bird mixing that may be difficult to track. For example, this could lead to higher network connectivity that may promote pathogen transmission, and to business models and practices that seek to generate margins from shortcuts or lower standards in relation to hygiene in bird handling or quality and health of birds traded.

Another factor of growing uncertainty is the entry of bigger corporate actors into the retail segment with their own wholesale procurement systems. Several players are recently pushing into the market with new kinds of modern branded retail outlets and home-delivery options. Major Indian corporations are also gearing up to supply more dressed and processed chicken through fully integrated PDNs from farm to fork and to increasingly shift to centralised slaughter and cold chain-based distribution and retail. So far, the wholesalers we spoke to did not seem existentially threatened by these, and neither by the slow increase in market share for dressed chilled and frozen broiler products that saw a boost in acceptance during the pandemic according to wholesalers. The demand for live broilers slaughtered on the spot remains strong and the wholesalers do not expect this to change soon. Some wholesalers, however, are concerned that the wholesale-cum-retail trade in live poultry may eventually be banned given its perceived public health threat, which may further the corporate control of the broiler meat market, as had been observed with other commodities:

*‘This business is a live market, but we don't know how many years it will remain a live market, isn't it? If dressed market will come, big companies will enter and the wholesale and retail sector will change to completely depend on them. Similarly, as you can't see the fresh milk vendors today, as we changed to pre-packed milk; likewise, the chicken industry will change'*.

However, while that will change potential epidemiological dynamics, such change does not necessarily mean increased food safety [47] and risks bringing other negative externalities of corporate concentration within the agri-food system [52]. Such changes may also have other effects for network configurations and associated epidemiological risks. In China's Guangxi, for example, poultry production companies established centralised trading platforms where birds from contracted farms are brought and reassorted according to weight and size specifications by clients, increasing the risks associated with the mixing of birds [6].

These insights call for targeted surveillance in accordance with these dynamics and emergent risks. Wholesale hubs of traders could be prioritised as potential reservoirs [49], with routine screening for avian influenza and antimicrobial resistance. Further, biosecurity practices might be incentivised through tiered regulations that certify established traders who adopt appropriate hygiene protocols, while informal traders could be offered training and subsidies [54]. Further engagement with broiler traders would be desirable, however, to explore co-developing and trialling workable mechanisms to make any surveillance and regulatory biosecurity interventions most effective and practically workable.

## 4. Conclusion

We have investigated the wholesale broiler trade from production sites to retail or restaurant outlets in Chennai. Our goal was to understand the PDN configurations and practices relevant for questions of how and where epidemiologically relevant risks could emerge. Thus, we provide an overview of the complex and dynamic configurations of Chennai-centric broiler PDNs which can support risk assessment and the design of targeted surveillance or mitigation approaches. Further, we situated the configuration of Chennai's broiler PDNs in an historical trajectory while anticipating future tendencies toward change that are likely significant for a dynamic approach towards understanding changing and emergent areas of risk.

While this study is exploratory, it points towards new ways of interrogating the market in its social and economic complexities, including the financial value that different actors extract from it. Our point is that the broiler market and PDN in and around Chennai, and other locations in India and elsewhere, need to be better understood and theorised in their entirety. This is an important area that merits more systemic and systematic social and economic research. We only make an initial attempt to link aspects of the structural functioning of the broiler market to the generation of potential epidemiological risks and suggest that there is promising potential for a better integration of social, economic and epidemiological investigation of livestock PDNs [26]. Prior work has already made the case for integrating economics and epidemiology, especially on the nexus of poverty and disease and by paying attention to what happens on the ground in terms of context and the constraints value chain actors face [25]. We contend, however, that the social and economic sciences hold wider repertoire than the conventional focus on economics and related behavioural incentives that might appear like a natural go-to approach in resonance with epidemiology for its relative ease of conceptual integration. Beyond such work, we suggest that there is important and necessary scope for a stronger engagement with social sciences—and social theory—for understanding markets and the practices of livestock value chain actors much more profoundly as historically, socially and culturally constituted, and shaped through different structural factors that together translate into value chain configurations and actors' practices [44, 55]. These, we suggest, cannot be adequately captured in terms of what shapes them through institutional economics and behavioural approaches. In other words, we believe the social sciences can play a greater role in understanding value chain dynamics and actors' practices beyond the contributions made to integrating economics and epidemiology.

More specifically, we suggest an interdisciplinary triangulation of approaches towards understanding livestock PDNs to contribute to veterinary epidemiology and One Health research more broadly, combining: (1) an understanding of the structure of markets and PDNs and the actors involved in them, (2) the capital and power that actors have to determine and negotiate their actual functioning, and profit margins and value extraction by involved actors, as inherent to the pursuits of political economy, economics and sociology, (3) the in-depth qualitative empirical study of actual experiences, perspectives and practices by actors as accessed through ethnographic approaches to immersive fieldwork based on participant observation, anthropologically conceived or otherwise, and (4) epidemiology proper towards understanding how actual production and distribution pathways in combination with their structural and practical characteristics generate specific risks. Finally, while here we intended to demonstrate the applied value of ethnographic methods to contribute to epidemiological research and surveillance strategies, an interdisciplinary engagement between epidemiology and social sciences cannot get around engaging with the critical conceptual contributions of the latter, which challenge the consensus derived within quantitative and positivist disciplinary approaches and introduce broader qualitative perspectives [56, 45, 46, 57, 58]. We suggest that integration of these approaches is an overdue innovation for the field of veterinary epidemiology and the pursuit of a vision of One Health which puts animal and human health firmly within the framework of the social and economic genesis of disease and its transmission. And in the same way as Barnett et al. [26] urge for social scientists to become part of interdisciplinary research teams, outbreak response teams should also include social scientists to interpret livestock traders' and other value chain actors' practices.

## Data Availability

The data that support the findings of this study are available upon reasonable request. The data are not publicly available due to ethical considerations.
